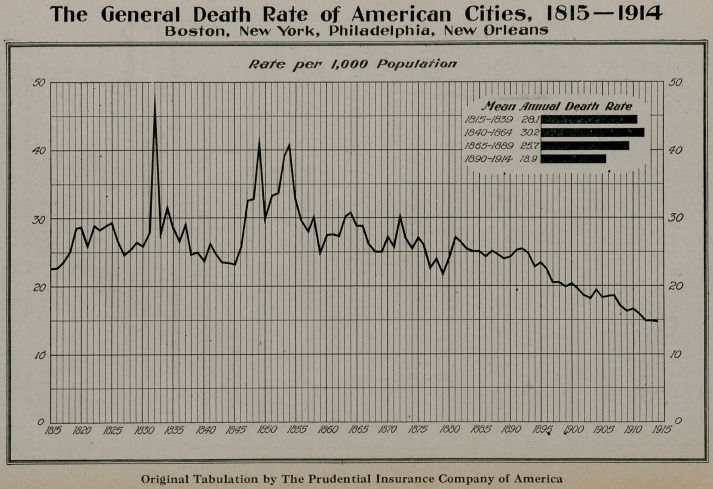# American Public Health

**Published:** 1915-10

**Authors:** 


**American Public Health.** Cuts loaned by Prudential Ins. Co. Data compiled by Frederick L. Hoffman, Statistician, Newark, N. J.

## Figures and Tables

**Figure f1:**
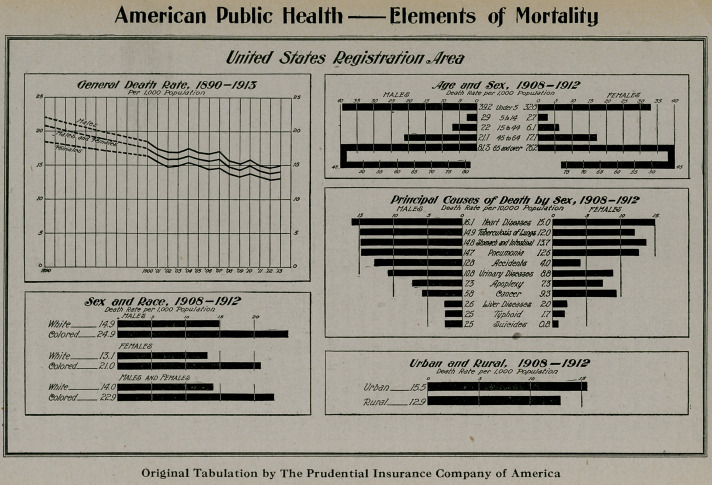


**Figure f2:**